# A novel management for postprostatectomy urinary incontinence: platelet-rich plasma urethral sphincter injection

**DOI:** 10.1038/s41598-021-84923-1

**Published:** 2021-03-08

**Authors:** Ping-Jui Lee, Yuan-Hong Jiang, Hann-Chorng Kuo

**Affiliations:** Department of Urology, Hualien Tzu Chi Hospital, Buddhist Tzu Chi Medical Foundation and Tzu Chi University, Hualien, Taiwan, ROC

**Keywords:** Urology, Prostate cancer

## Abstract

Platelet-rich plasma (PRP) is the most innovative blood-derived product used in regenerative medicine. We aimed to investigate the therapeutic efficacy of PRP urethral sphincter injection for the management of postprostatectomy incontinence (PPI). In total, 28 PPI patients with a mean age of 71.8 ± 8.9 years were prospectively enrolled. They received four PRP urethral sphincter injections each month. The clinical outcomes were assessed 3 months after the fourth injection as posttreatment Global Response Assessment (GRA) score, the newly designed visual analogue scale of stress urinary incontinence (VAS of SUI), and in urodynamic parameters. After injections, the posttreatment median GRA with quartiles was 2.0 (1.0, 2.0). Overall, six (21.4%) patients achieved complete continence and pad-free status, 20 (71.4%) achieved successful outcome (GRA score ≥ 2), and 26 (92.9%) showed clinical improvement (GRA score ≥ 1). The VAS of SUI significantly improved from 6.5 (5.0, 8.0) to 3.5 (2.0–5.8) (p < 0.001) as well as abdominal leak point pressure, from 57.5 (50.0, 115.0) to 126.0 (68.3, 150.0), (p = 0.004). After repeated PRP urethral sphincter injections, the SUI severity reduced significantly with high success rates. There was no major adverse event, except three patients with mild hematuria and micturition pain. In conclusion, PRP urethral sphincter injection is safe and effective as a novel management of PPI.

## Introduction

Stress urinary incontinence (SUI) can occur in men after radical prostatectomy (RP), referred to as postprostatectomy incontinence (PPI). PPI can cause significant distress and affect the quality of life. With increased number of surgeries for prostate cancer, there has been a concomitant increase in PPI prevalence^1^.

The initial management of PPI includes lifestyle interventions, pelvic floor muscle training, bladder retraining, pharmacotherapy, and combination therapy^2^. After the failure of initial conservative treatment, surgical interventions such as injectable bulking agents, male slings, and artificial urinary sphincters (AUS) to restore urethral competence have been suggested^3,4^. However, patients with PPI may hesitate to undergo surgery when their symptoms are mild to moderate. An effective, simpler, and less invasive approach is warranted for patients with PPI who are reluctant or contraindicated to undergo invasive surgical interventions.

Platelet-rich plasma (PRP) is an autologous blood-derived product obtained directly from patients’ peripheral blood. It comprises a high concentration of platelets and a pool of cytokines, chemokines, and growth factors and is known to contribute to regeneration^5^. The wide range of secreted proteins and growth factors within the α-granules in platelets promotes not only thrombosis and hemostasis but also chemotaxis, cellular proliferation, differentiation, angiogenesis, vascular modeling, and immune interactions^6,7^. Therefore, PRP has emerged as an innovative and versatile formulation in the treatment of osteoarthritis, chondral pathologies, nonunion fractures, tendinopathies, muscle strains, and neuropathies^8^.

The potential role of PRP in treating SUI caused by a defect in the pubourethral ligament was first proposed in 2016^9^. In 2019, a significant increase in the leak point pressure after PRP application in the transected pubourethral ligaments in mice was also reported^10^. Accordingly, PRP urethral sphincter injection may have the potential to treat PPI. Moreover, compared to the use of male sling and AUS for PPI treatment, autologous PRP urethral sphincter injection is less invasive and superior than the implantation of synthetic materials with respect to surgical burden and adverse effects. Therefore, this prospective, single-center, uncontrolled, and proof-of-concept study aimed to investigate the therapeutic efficacy of autologous PRP urethral sphincter injection for the management of PPI refractory to conservative treatment.

## Results

A total of 28 patients with a mean age of 71.8 ± 8.9 years and a mean SUI duration of 48.7 ± 42.7 (range 12–180) months were enrolled in this study. Open, laparoscopic, and robotic-assisted RP were performed in eight, five, and 15 patients, respectively. Ten (35.7%) patients presented with VAS of SUI 1–5 (i.e., Stamey Grade 1 SUI), 16 (57.1%) patients presented with VAS of SUI 6–9 (i.e., Stamey Grade 2 SUI), and two (7.1%) patients presented with VAS of SUI 10 (i.e., Stamey Grade 3 SUI). Pad protection was necessary in all patients before treatment. The mean follow-up time after the entire treatment course was 13.4 ± 5.7 months.

The posttreatment median GRA with quartiles was 2.0 (1.0, 2.0) (mean with standard deviations: 1.9 ± 0.8, range: 0–3, (Table 1). Overall, 26 (92.9%) experienced clinical improvement (GRA score ≥ 1), and 2 (7.1%) revealed no improvement (GRA score = 0). Among patients with improvement, 20 (71.4%) achieved a successful outcome (GRA score ≥ 2). Finally, six (21.4%) patients achieved complete continence and pad-free status. The other six (21.4%) patients confirmed the clinical therapeutic effect and requested for the extra injection course.

**Table 1:** Clinical outcomes and change of urodynamic parameters after platelet-rich plasma urethral sphincter injection treatments.

	Total (n = 28)		
**Primary endpoints**			
GRA score, median (Q1, Q3)	2.0 (1.0, 2.0)		
GRA score = 0	2 (7.1%)		
GRA score = 1	6 (21.4%)		
GRA score = 2	14 (50.0%)		
GRA score = 3	6 (21.4%)		
Improvement (GRA score ≥ 1)	26 (92.9%)		
Successful outcome (GRA score ≥ 2)	20 (71.4%)		

	Baseline Median (Q1, Q3)	After treatment Median (Q1, Q3)	P-value
**Secondary endpoints**			
VAS of SUI	6.5 (5.0, 8.0)	3.5 (2.0–5.8)	< 0.001
**VUDS parameters**			
CBC (mL)	336.5 (226.8, 390.5)	307.0 (193.5, 383.0)	0.640
Vol (mL)	302.0 (187.5, 378.5)	255.0 (133.5, 359.5)	0.719
PVR (mL)	0 (0, 12.5)	0 (0, 4.5)	0.780
Qmax (mL/s)	10.5 (7.0, 14.0)	12.0 (6.0, 15.5)	0.740
Pdet (cmH_2_O)	15 (4.8, 20.0)	13.0 (6.0, 20.0)	0.868
BOOI	− 5.6 (− 22.5, 3.8)	− 11.0 (− 16.5, 0.5)	0.968
ALPP (cmH_2_O)^§^	57.5 (50.0, 115.0)	126.0 (68.3, 150.0)	0.004

*ALPP* abdominal leak point pressure; *BOOI* bladder outlet obstruction index; *CBC* cystometric bladder capacity; *GRA* global response assessment; *Pdet* detrusor pressure at maximum flow rate; *PVR* postvoid residual; *Qmax* maximum flow rate; *VAS of SUI* visual analogue scale of stress urinary incontinence severity; *Vol* voided volume; *VUDS* videourodynamic study.

^§^Nine patients in the pre-treatment stress test, and fourteen patients in post-treatment stress tests during VUDS did not demonstrate urinary leakage (i.e., no valuable ALPP), and the ALPP values of these patients were excluded for analysis.

**Figure 1:**
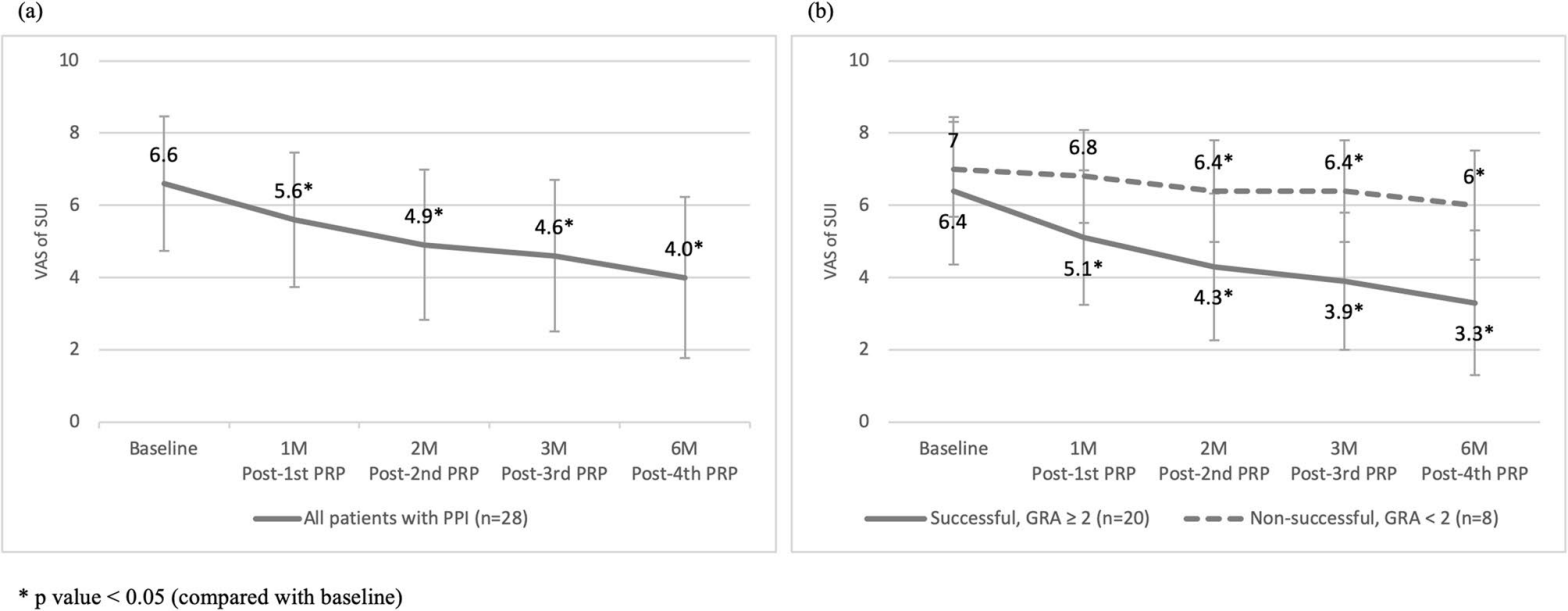
The changes in stress urinary incontinence (SUI) severity as per the visual analogue scale of SUI after autologous platelet-rich plasma urethral sphincter injections (**a**) in all patients; (**b**) Subgroup analysis by outcome.

After four PRP urethral sphincter injections, the SUI severity reduced significantly (VAS of SUI from 6.5 (5.0, 8.0) to 3.5 (2.0–5.8) (p < 0.001). (Table 1, Fig. 1a) Significant therapeutic effects were observed immediately after the first injection treatment and persisted throughout the study period. Similarly, the prominent results were achieved in both successful and non-successful subgroups. The changes in the VAS of SUI after treatment were significant (successful and non-successful, p = 0.003 and 0.019, respectively; Fig. 1b). None of the VUDS parameters changed significantly except for ALPP, which increased from 57.5 (50.0, 115.0) to 126.0 (68.3, 150.0) cmH_2_O (p = 0.004) (Table 1). PRP urethral sphincter injection therapy for PPI did not exert any negative effects on lower urinary tract function.

The logistic regression model presented in Table 2 revealed that factors such as the age, SUI duration, initial severity of SUI, and lower urinary tract function did not significantly impact the patient’s successful outcomes.

**Table 2:** Logistic regression analysis for predictors of successful outcome.

	Odds ratio (95% CI)	P value
Age	0.981 (0.892–1.080)	0.701
SUI duration	1.012 (0.982–1.043)	0.424
**Initial SUI severity**		
VAS of SUI 1–5	Reference	–
VAS of SUI 6–10	0.500 (0.080–3.127)	0.459
**Baseline VUDS parameters**		
CBC	1.003 (0.996–1.011)	0.342
Vol	1.001 (0.994–1.008)	0.774
PVR	1.008 (0.990–1.028)	0.379
Qmax	1.031 (0.869–1.223)	0.727
Pdet	0.943 (0.856–1.038)	0.231
BOOI	0.978 (0.918–1.042)	0.499
ALPP	1.048 (0.983–1.117)	0.149

*ALPP* abdominal leak point pressure; *BOOI* bladder outlet obstruction index; *CBC* cystometric bladder capacity; *Pdet* detrusor pressure at maximum flow rate; *PVR* postvoid residual; *Qmax* maximum flow rate; *VAS of SUI* visual analogue scale of stress urinary incontinence severity; *Vol* voided volume; *VUDS* videourodynamic study.

Perioperatively, there was no severe adverse events or complications such as acute urinary retention or urinary tract infection developed, and none of the participants experienced deterioration in terms of the SUI severity after PRP urethral sphincter injection. Mild hematuria and micturition pain were noted in three (10.7%) patients and resolved after the administration of conservative treatment.

## Discussion

This is the first prospective study to demonstrate the efficacy and safety of repeated PRP urethral sphincter injection treatment on PPI with significant clinical and urodynamic evidence. After repeated PRP urethral sphincter injections, the SUI severity reduced significantly with high success and improvement rates regardless of the baseline characteristics. Moreover, ALPP, indicating urethral resistance, significantly improved posttreatment. More importantly, this injection procedure is safe and does not have major adverse effects even after repeated procedures. PRP urethral sphincter injection has the potential to be a novel approach for the management of PPI.

The treatment of medical refractory PPI is generally based on invasive surgical interventions. According to the recent systemic review and recommendations of the International Consultation on Incontinence, AUS is the most effective treatment option for moderate-to-severe PPI with a success rate of 59–90%^3,11^. However, with the concerns of costs, complications, and unneglectable revision rate, AUS is not suitable for all patients. The use of male sling is a viable alternative to AUS in patients with mild-to-moderate PPI. However, the success rate of male sling widely varies (9–87%) with different kinds of slings and lack of long-term data. Injectable bulking agents are an inferior option because of deterioration of effect over time, and very low cure rates^11^. In our study, after the administration of PRP urethral sphincter injections for PPI, 21.4% patients achieved continence and pad-free status, and most patients (92.9%) had clinical improvement with minimal surgical burden and complications. With the concerns of efficacy and safety, PRP urethral sphincter injection may play a certain role in the future treatment of PPI.

In recent years, cellular therapy for the treatment of SUI has been generally shown to be feasible and safe; however, it is limited by modest efficacy^12^. PRP, another mainstream technology in regenerative therapy, was proven to promote the regeneration of peripheral nerves by secreting large quantities of growth factors and few neurotrophic factors. Platelet-derived growth factor protects neurons by suppressing the N-methyl-D-aspartic acid-evoked current and translocating the glutamate transporter to the cell membrane^13^, transforming growth factor-β1 increases the synthesis of extracellular matrix components and promotes neuron survival^14^, insulin-like growth factor-I enhances the outgrowth of corticospinal motor neuron axons in vitro^15^, brain-derived neurotrophic factors play an early anti-inflammatory role and promote axonal sprouting and functional recovery after spinal cord injury^16^. However, thus far, PRP has not been used in patients with PPI. In this study, by the role of regenerative capacity, we applied repeated PRP urethral sphincter injections to resume and increase urethral competence in patients with PPI. It is hypothesized that the deficient urethral sphincter function improves after regenerated innervation and increased striated muscle cell volume via repeated PRP urethral sphincter injections into the external urethral sphincter.

Bottegoni et al. studied 60 patients with symptomatic knee osteoarthritis receiving homologous intra-articular PRP injections and observed a less favorable clinical outcome in patients aged ≥ 80 years versus those aged 65–79 years^17^. In this study, the influence of age on clinical outcomes was not significant. Moreover, we did not identify significant correlations between SUI duration, initial SUI severity, lower urinary tract function and clinical outcomes. This study with few cases revealed that most patients benefited from urethral injection of PRP regardless of the baseline characteristics.

After PRP urethral sphincter injections, the noted increase in ALPP indicates an increase in urethral resistance. Similar results have been reported by Lee et al. stating that injection of placebo saline was followed by a temporary improvement similar to bulking agents injection and have raised questions regarding the mechanism involved in these beneficial effects^18^. In this study, the clinical therapeutic effect could not be observed until days after the injection and did not disappear with time. It is less likely that the PRP injection has a bulking effect on PPI. However, whether this effect is a result of the true PRP effects or the effect caused by the injection requires further and longer investigation. Additionally, this repeated PRP urethral sphincter injection therapy for PPI did not cause negative effects on lower urinary tract function.

There are some limitations in this study. First, there was a lack of a placebo-controlled group because sham interventions are prone to cause ethical conflicts and moral concerns in clinicians. Second, the standard dose and preparation of PRP, the injection techniques, and the ideal regimen of repeated injection have not been established. Third, there is no direct evidence to validate the supposed regeneration of sphincter innervation and increase in striated muscle cell mass through repeat PRP urethral sphincter injections. Finally, the SUI severity score used in the present study has not been well validated, although it is established based on the previously well-accepted Stamey grade of SUI. In contrast to the cumbersome pad tests, this new classification of SUI severity may provide clinical physicians with an easier tool for assessing the SUI severity in clinical practice by taking the history of situation of SUI, frequency of SUI, and status of pad protection.

## Methods

### Patients

This prospective study was conducted from September 2018 to April 2020. This study was approved by the Institutional Review Board and Ethics Committee of the Hualien Tzu Chi Hospital, Buddhist Tzu Chi Medical Foundation, Taiwan (IRB NO. 107-231-A). All methods were performed in accordance with the relevant guidelines and regulations. Patients were informed about the study rationale and procedures; written informed consent was obtained from all patients prior to enrollment and treatment. All eligible patients had de novo SUI after RP and were refractory to conservative treatment including lifestyle modifications, pelvic floor muscle training, bladder retraining, and pharmacotherapy for at least 12 months. Furthermore, all patients underwent videourodynamic studies (VUDS) and abdominal leak point pressure (ALPP) study; urethral incompetence was diagnosed as the etiology of PPI. Patients with large post-void residual (PVR) volumes of ≥ 150 ml, known platelet dysfunction, anticoagulant use, critical thrombocytopenia, hypofibrinogenemia, hemodynamic instability, sepsis, acute or chronic infections, chronic liver disease, and known malignancy were excluded.

### Baseline clinical assessment

The clinical data of age, monthly duration of SUI, systemic comorbidities, surgical type of RP, and total follow-up time after treatment (in months) were collected. We determined the severity of PPI by using visual analogue scale (VAS) of SUI (Table 3), which was newly designed according to the previously reported Stamey SUI grading system^19^. The VAS of SUI was in the range of 0–10, indicating complete continence to total urinary incontinence. All patients underwent comprehensive VUDS prior to treatment. The procedures of VUDS, ALPP and terminology of the urodynamic parameters were in accordance with the recommendations of the International Continence Society^20^. Urodynamic parameters, including cystometric bladder capacity (CBC), voided volume (Vol), PVR, maximum flow rate (Qmax), detrusor pressure at Qmax (Pdet), and ALPP were recorded.

**Table 3:** Visual analogue scale (VAS) for assessment of stress urinary incontinence (SUI).

VAS of SUI	Corresponding Stamey grade of SUI	Situation of SUI	Frequency of SUI (on situation)	Pad protection
0	0	Complete dryness	0	No
1	1	Any mild or severe straining situation	1 episode per days to weeks	No/Yes
2	1	Heavy straining/squatting	1 episode per day	No/Yes
3	1-2	Coughing/sneezing/laughing/nighttime/change position	1 episode per day	No/Yes
4	1	Heavy straining/squatting	> 1 episode per day	No/Yes
5	1-2	Coughing/sneezing/laughing/nighttime/change position	> 1 episode per day	No/Yes
6	2	Walking	< 50% situation (every day) & > 1 episode per day	Yes
7	2	Very mild movement/change position	< 50% situation (every day) & > 1 episode per day	Yes
8	2	Walking	≥ 50% situation (every day) & > 1 episode per day	Yes
9	2	Very mild movement/change position	≥ 50% situation (every day) & > 1 episode per day	Yes
10	3	Any condition; persistent	All the time	Yes

### The preparation of PRP and procedure of urethral sphincter injection

A total of 50 ml of whole blood was withdrawn from the peripheral vein, followed by two centrifugation steps at a licensed laboratory for the preparation of PRP. Initially, a soft spin at 200×*g* for 20 min at 20 °C was performed to distinguish the plasma and erythrocyte layers. The upper plasma layer was aseptically collected and subjected to a second hard spin at 2000×*g* for 20 min at 20 °C. In total, 5 ml of PRP (approximately 2.5–fivefold increased platelet concentration than that in the peripheral blood) was obtained^21^.

**Figure 2:**
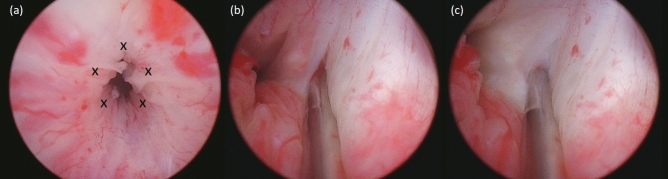
Transurethral urethral sphincter injection of platelet-rich plasma in patients with post-prostatectomy incontinence (**a**) Crosses indicate injection sites; (**b**) The needle was injected at a depth of 0.5 cm; (**c**) Swelling of the urethral surface after the injection.

All injections were transurethrally administered using a rigid cystoscopic injection instrument (23 Fr; Richard Wolf, Knittlingen, Germany) under intravenous general anesthesia. Perioperatively, the needle was inserted into the external urethral sphincter circumferentially with five injection sites at 2, 5, 7, 10, and 12 o’clock positions (Fig. 2). The swelling of the urethral surface was identified after each injection to confirm the injection effects. After the injections, patients did not receive urethral catheterization and were discharged on the next day if no complication developed. The procedures were repeated every month, with a total of four injections.

### Outcome measurements

The primary treatment outcomes were assessed using the Global Response Assessment (GRA) score (categorized into − 3, − 2, − 1, 0, 1, 2, and 3, indicating markedly worse to markedly improved status)^22^ and VAS of SUI reported by the patients themselves posttreatment. All patients were sequentially interviewed and requested to complete questionnaires at baseline, 1 month after each urethral injection (first three times) of PRP (i.e., just before the next injection), and 3 months after the fourth injection. Repeated VUDS and ALPP studies were performed at 3 months after the fourth injection to evaluate any changes in the lower urinary tract functions.

The primary endpoint of this study was posttreatment GRA score after four PRP urethra sphincter injections. Successful outcome was defined as GRA score ≥ 2 (moderate and marked improved), and GRA score ≥ 1 was considered as having clinical improvement. The secondary endpoints included changes in the VAS of SUI and changes in urodynamic parameters from baseline to 3 months after the fourth PRP urethra sphincter injection.

### Statistical analysis

The categorical data are shown as numbers and percentages, and the continuous variables are presented as means with standard deviations and median with quartiles. The Wilcoxon’s signed-rank test was used to distinguish the difference of variables in patients between baseline and after treatment. The Wilcoxon’s rank sum test was used for statistical comparisons of variables in between-subgroup analysis. The logistic regression analysis was used to predict factors associated with successful outcomes. SPSS version 25.0 (Inc., Chicago, IL, USA) was used for statistical analyses. P < 0.05 was considered statistically significant.

## Conclusions

PRP urethral sphincter injection for the treatment of PPI is safe, minimally invasive, and effective with significant clinical and urodynamic evidence. It has the potential to be a novel approach for the treatment of PPI.

## Data Availability

To protect patient privacy and comply with relevant regulations, identified data are unavailable. Requests from qualified researchers with appropriate ethics board approvals and relevant data use agreements for de-identified data will be available.
